# Shifts in bacterial community composition during symbiotic seed germination of a terrestrial orchid and effects on protocorm development

**DOI:** 10.1128/spectrum.02185-24

**Published:** 2024-11-14

**Authors:** Zeyu Zhao, Luna Yang, Yaoyao Wang, Xin Qian, Gang Ding, Hans Jacquemyn, Xiaoke Xing

**Affiliations:** 1 State Key Laboratory for Quality Ensurance and Sustainable Use of Dao-di Herbs, Institute of Medicinal Plant Development, Chinese Academy of Medical Sciences and Peking Union Medical College, Beijing, China; 2 Department of Biology, Plant Conservation and Population Biology, Katholieke Universiteit Leuven, Leuven, Belgium; Instituto de Ecología, A.C. (INECOL), Pátzcuaro, Michoacán, Mexico

**Keywords:** endophytic microbiome, *Pseudomonas*, growth promotion, *Gymnadenia conopsea*

## Abstract

Fungi and bacteria often occupy very similar niches; they interact closely with each other, and bacteria can provide direct or indirect benefits to plants that form mutualistic interactions with fungi. In orchids, successful seed germination largely depends on compatible mycorrhizal fungi, but whether and how bacteria contribute to seed germination and protocorm development remains largely unknown. Here, we performed field and laboratory experiments to assess the potential role of bacteria in mediating seed germination and protocorm development in the terrestrial orchid *Gymnadenia conopsea*. Our results suggested that bacterial and fungal communities differ between developmental stages in the germination process. The diversity of bacterial and fungal communities and their interaction network in germinating seeds (Stage 1) differed significantly from those in later developmental stages (Stages 2–5). *Pseudomonas* gradually became the dominant bacterial group as the protocorms matured and showed a positive association with Ceratobasidiaceae fungi. Seed germination tests *in vitro* demonstrated that co-inoculation of *Ceratobasidium* sp. GS2 with *Pseudomonas* isolates significantly improved protocorm growth and development, suggesting that the observed increase in *Pseudomonas* abundance during protocorm development directly or indirectly improves the growth of germinating seeds. Overall, our findings indicate that bacteria may exert non-negligible effects on seed germination of orchids and, therefore, offer valuable perspectives for future strategies for conservation and cultivating orchid species.

**IMPORTANCE:**

It is well known that orchid seeds depend on mycorrhizal fungi to supply the necessary nutrients that support germination in natural environments. Apart from fungi, bacteria may also be involved in the germination process of orchid seeds, but so far, their role has not been intensively studied. This research provides evidence that bacterial community composition changes during seed germination of the terrestrial orchid *Gymnadenia conopsea*. Interestingly, *in vitro* experiments showed that *Pseudomonas* spp., which were the most dominant bacteria in the later germination stages, improved protocorm growth. These results suggest that bacteria contribute to the germination of orchid seeds, which may open new perspectives to apply bacteria as a biofertilizer in the introduction and restoration of *G*. *conopsea* populations.

## INTRODUCTION

Symbiosis is one of the most ubiquitous interactions between plants and fungi in global ecosystems (1, 2). During the long process of evolution from an aquatic to a terrestrial environment, plant-beneficial fungi have played an important role in plants adapting to novel ecological environments (3) as they have essential biological and ecological functions, such as enhancing nutrient availability, providing growth hormones, increasing plant resistance against both biotic and abiotic stresses, and improving soil structure and fertility (1, 4–6). Harnessing the potential of plant-beneficial fungi holds great promise for revolutionizing plant cultivation techniques by improving plant growth conditions, enhancing productivity, and increasing pathogenic resistance.

Bacteria commonly live together with fungi and may benefit their plant hosts directly or indirectly (7, 8). The symbiotic association with bacteria has been a fundamental prerequisite in plants since their terrestrialization approximately 400 million years ago (9). Bacteria that confer direct benefits to plant hosts are known as plant growth-promoting bacteria (PGPB) (10). These bacteria are ubiquitous in the soil and within plant tissues and promote plant growth and improve plant defenses (10–12). In plants that form interactions with fungi, a specific group of bacteria, the so-called mycorrhiza helper bacteria (MHB), can provide indirect benefits to plants as they enhance mycorrhizal growth and nutrient uptake by the fungus and the plant, improve soil conductance, aid against specific pathogens, and help promote defense mechanisms (8, 12, 13).

Most known MHB belong to Proteobacteria, Actinobacteria, Firmicutes, and Bacteroidetes (10, 14). They are naturally present in the soil and form complex interactions with fungi as soon as roots develop. In most cases, endophytic bacterial communities also change with the host plant’s phenological development, hence, pronounced differences can be found between phenological stages (15, 16). Moreover, in many cases, it can be expected that the recruitment of endophytic bacteria is mediated by the presence of fungal symbionts (17, 18). For example, in alpine bistort (*Bistorta vivipara*, Polygonaceae), the spatial structure of root-associated bacterial microbiomes was significantly related to mycorrhizal network structure (19). Similarly, arbuscular mycorrhizal fungi (AMF) may play a role in the recruitment of endophytic bacterial communities by host plant roots (20).

With more than 28,000 species, the Orchidaceae is one of the most prominent families of flowering plants. The reproduction of orchids depends on the availability of suitable pollinators and mycorrhizal fungi (2, 21). Orchid seeds are tiny and lack an endosperm, and therefore depend on mycorrhizal fungi to supply the nutrients supporting their germination in natural environments (2, 22). Isolation and screening of compatible fungal strains from protocorms or roots of adult plants and their further use in promoting seed germination have been proven effective for population recovery and germplasm conservation of rare and endangered orchids (23–27). Although germination of orchid seeds largely depends on compatible mycorrhizal fungi, several studies have indicated that bacteria isolated from orchid roots have the potential to promote seed germination and subsequent seedling growth, and hence may play a direct or indirect role in the germination process of orchid seeds (9, 28–32). However, much remains unknown about how bacteria assemble during orchid seed germination and their contribution to protocorm development. Bridging this knowledge gap is crucial for a better understanding of orchid seed germination in natural environments and may shed light on microbial community dynamics for potential use in orchid cultivation.

To improve our understanding of the diversity and composition of bacterial communities associated with germinating orchid seeds, we performed field and laboratory experiments using the seeds of *Gymnadenia conopsea*. This perennial herbaceous orchid is widely distributed throughout Europe and Asia’s temperate and subtropical zones (33). Specifically, we performed *in situ* and *in vitro* seed germination experiments to (i) investigate how bacterial diversity and community composition change during the germination process, (ii) determine whether germinating seeds of *G. conopsea* recruit beneficial bacteria from their surroundings, and (iii) assess whether these bacteria can promote orchid seed germination or protocorm development. Changes in bacterial communities were investigated for different stages of seed germination using high-throughput sequencing. Subsequently, bacterial strains were isolated from protocorms obtained in the field, and *in vitro* seed germination experiments were conducted to investigate the potential of bacteria to stimulate seed germination and promote protocorm growth.

## MATERIALS AND METHODS

### Study species

*Gymnadenia conopsea* is a widespread terrestrial orchid that occurs in most of Europe and Asia. It is a mycorrhizal generalist that is associated with a wide variety of fungi (33–36). In recent years, the species has suffered dramatic declines in distribution and abundance due to overgrazing, over-collection, and habitat loss (37). As a result, *G. conopsea* has been listed as a class II protected plant in China’s List of National Key Protected Wild Plants (National Forestry and Grassland Administration and Ministry of Agriculture and Rural Affairs, 2021).

### Study site and seed collection

The study was conducted at the Birishen Mountain Scenic Spot in Bayi Town, Nyingchi County, Tibet, China (29°45′54″N, 94°24′22″E). All research was conducted in a meadow plot at the edge of a forest 3,690 m above sea level. Twenty individuals of *G. conopsea* were detected within this plot. Seeds of *G. conopsea* were collected in mid-August 2020. The seed capsules were detached from their stalks, air-dried, and stored in paper bags at 4°C.

### *In situ* seed germination and sample collection

Previous research has shown that one fungal strain, *Ceratobasidium* sp. GS2 (NCBI accession number OK655751.1), which was isolated from the roots of adult *G. conopsea* plants, could support the germination of *G. conopsea* seeds under laboratory and natural conditions (37–39). *Ceratobasidium* sp. GS2 was cultured on potato dextrose agar (PDA) in 9-cm Petri dishes under 25°C in the dark for 1 week and then used for inoculation. The fungal inoculum was prepared as follows: a substrate consisting of wheat bran (800 g) and leaf fragments (200 g) was thoroughly mixed while keeping the final water content around 60%. The substrate was then put into 500-mL wide-mouthed bottles up to 3 cm below the bottleneck and was sterilized at 121°C for 4 hours. Each bottle was inoculated with two 1 × 1 cm plugs of the fungal strain from fresh cultures. Subsequently, the bottles were stored in the dark at 25°C for nearly 2 months. After hyphae had colonized the whole substrate in each bottle, the substrates were used as fungal inoculum for *in situ* seed germination experiments.

In April 2021, we performed *in situ* seed germination experiments within the same plot. Three hundred seed packets were buried in a 400-m^2^ plot. Seed packets were buried at least 2 m apart from adult *G. conopsea* plants to avoid the impact of rhizosphere microbes. One-third of the packets (*n* = 100) contained approximately 150 seeds and 2 g of substrate with fungal inoculum. Another hundred seed packets were filled with seeds and sterilized substrate, while the remaining 100 seed packets only contained seeds and served as control. All seed packets were buried in triplets (one seed packet for each treatment) at 20-cm intervals in the soil to a 10- to 13-cm depth. In September 2022 and June 2023, 50 seed packets from each experimental group were retrieved from the soil. The seed packets were kept humid and stored in an ice box until reaching the lab. Subsequently, the seed packets were examined for the presence of germinating seeds and protocorms using light microscopy.

**Fig 1:**
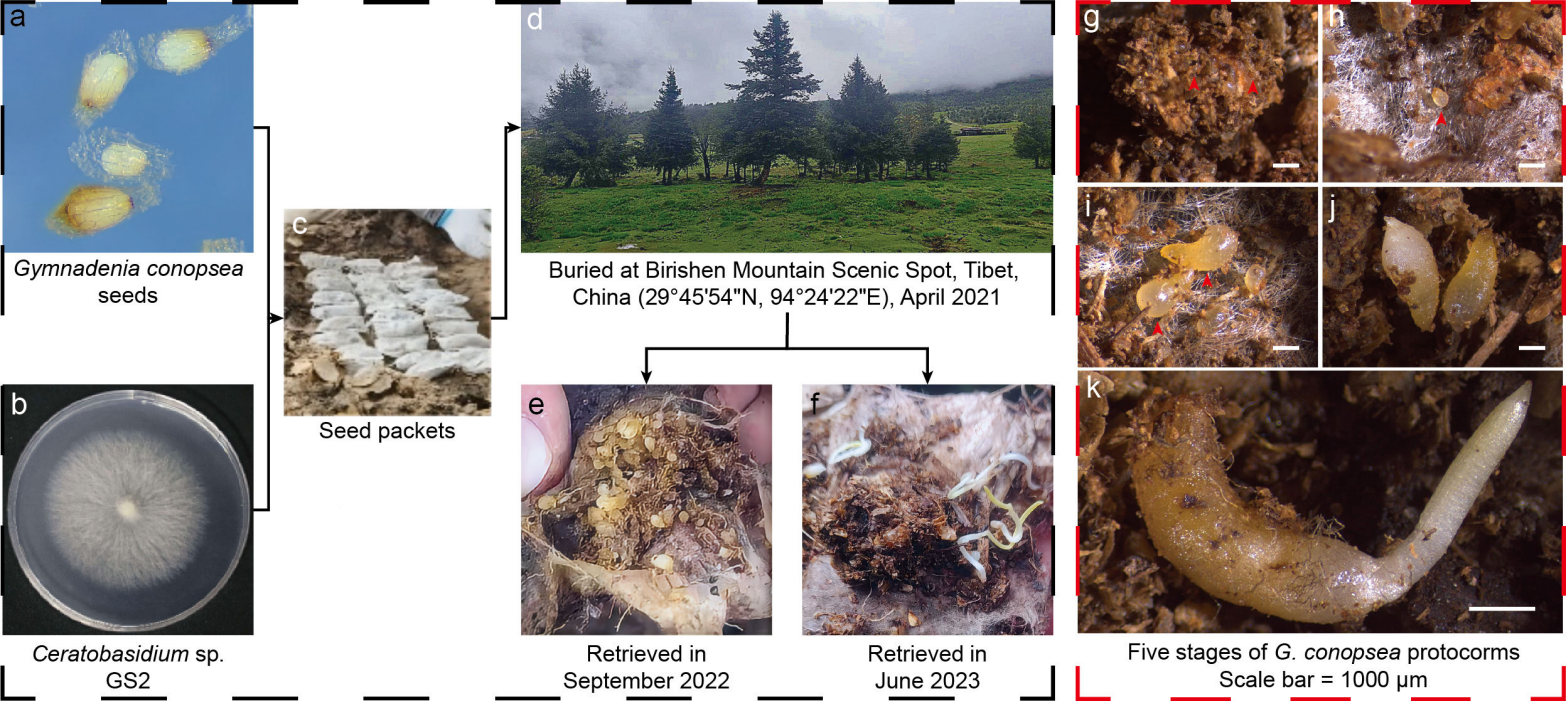
Overview of the *in situ* germination experiment and the different developmental stages in the germination process of the terrestrial orchid *Gymnadenia conopsea* seeds. The frame with the black dotted lines indicates the experimental setup *in situ*. In contrast, the pictures in the red dotted line frame represent protocorms at different developmental stages observed under a microscope. (**a**) Morphology of the *G. conopsea* seeds; (**b**) colony of the *Ceratobasidium* sp. GS2; (**c**) seed packets; (**d**) habitat of the experimental site; (**e and f**) retrieved seed packets; (g–k) germination process of *G. conopsea* seeds (from germination Stage 1 to 5) (scale bar = 1,000 µm).

Because no seed germinated in the control and the group with sterilized substrate, we only collected protocorms from the inoculated group. Five distinct stages in the germination process were distinguished: Stage 1—embryo imbibition; Stage 2**—**rupture of the testa; Stage 3**—**formation of the protomeristem; Stage 4**—**emergence of the first genuine leaf; and Stage 5**—**elongation of the first genuine leaf (40) (Fig. 1). For each germination stage, 10 individuals were selected randomly from different seed packets, and each individual was regarded as one sample. Fifty samples were collected and stored at −80°C for subsequent molecular analysis.

### DNA extraction and 16S rRNA sequencing

The seeds and protocorms were washed under tap water for 10 minutes and were surface sterilized by immersion in 75% ethanol for 10 seconds, 3.5% (vol/vol) NaOCl for 30 seconds, 75% ethanol for 10 seconds, and rinsed with sterile water three times. DNA extraction was performed using the CTAB rapid genome extraction kit-DN14 (Aidlab Biotechnologies, Beijing, China) following the manufacturer’s instructions. DNA quality and quantity were evaluated using agarose gel electrophoresis (1%) and a NanoDrop 2000 (Thermo Fisher Scientific, USA). To create the library, the DNA samples must have a total DNA concentration of over 50 ng/µL, A260/A280 > 1.8, and A260/A230 > 2.0.

The bacterial and fungal communities were analyzed by sequencing the 16S and ITS2 rRNA genes. The primer pair 27F (5′-TACGGYTACCTTGTTACGACTT-3′) and 1492R (5′-AGAGTTTGATCMTGGCTCAG-3′) was used for amplifying the bacterial 16S rRNA sequence, while the primer pair ITS86F (5′-GTGAATCATCGAATCTTTGAA-3′) and ITS4 (5′-TCCTCCGCTTATTGATATGC-3′) was employed to amplify the fungal ITS2 region. PCR conditions were as follows: initial denaturation of 2 minutes at 95°C, 25 cycles of denaturation at 98°C for 10 seconds, annealing for 30 seconds at 55°C, extension for 1.5 minutes at 72°C, and a final extension of 10 minutes at 72°C. PCR products were quantified according to the Qubit value, and their concentrations were adjusted. The bacterial and fungal amplicons were sequenced using the Sequel II platform (PacBio, USA) and the Illumina NovaSeq 6000 platform. The number of reads for each sample was adjusted according to the rarefaction curve to exhaust the diversity in samples and maximize the recovery of unique sequences.

### Microbiome assembly and annotation

The PacBio subreads data were exported as CCS files, and Lima version 1.7.0 was used to identify CCS raw reads. Simultaneously, the Illumina platform data underwent preprocessing using Trimmomatic v0.33 software to filter sequenced raw reads. Cutadapt version 2.7 was used to identify and remove primer sequences, ensuring clean CCS reads that do not contain primer sequences (41). Finally, QIIME 2 (version 2020.6, https://qiime2.org/) was used to analyze these clean reads (42). The 16S and ITS2 amplicons were then analyzed to amplicon sequence variants (ASVs) using DADA2 within the QIIME 2 plugin (43). ASVs with less than 0.005% of all sequence numbers were excluded. Taxonomic assignment of bacterial and fungal ASVs was performed using a sklearn-based Naive Bayes classifier plugin against the SILVA 138 99% 16S full-length and UNITE database. ASVs that were annotated as mitochondria and chloroplasts were filtered out from the data set.

### Bioinformatic analysis

To evaluate microbial diversity, we computed α-diversity (Shannon-Wiener index) and phylogenetic diversity (PD) using QIIME2. We statistically compared diversity differences among germination stages using a Kruskal-Wallis test with Dunn’s multiple comparisons test (*n* = 10). Finally, GraphPad Prism was used to generate box plots illustrating variation in diversity across germination stages. To assess variation in bacterial community composition, we performed a principal coordinate analysis (PCoA) using the Bray-Curtis distance measure. Permutational multivariate analysis of variance (PERMANOVA) was conducted using the “adonis” function in the vegan package in R (44) to see whether bacterial community composition differed among germination stages. All graphs were drawn using the ggplot2 package in R (45). The Venn diagram based on bacterial and fungal ASV was drawn using the online software jvenn (46).

Bacterial and fungal biomarkers were identified using Linear discriminant analysis Effect Size (LEfSe) (47), with an LDA threshold score of 4.0 and a significance level of *P* < 0.05. In addition, the “IndVal” function from the labdsv package in R was used for Dufrêne-Legendre indicator species analysis, with indicator values > 0.5 and a significance level of *P* < 0.05 as thresholds (48, 49).

Co-occurrence networks were constructed using fungal and bacterial genera that were detected in more than 20% of the samples. The network was built using the “graph.adjacency” function from the igraph package in R (50). The Gephi (v0.9.5) platform was used to visualize the networks.

The functional abundance of the samples was predicted using QIIME2’s PICRUSt2 plugin. First, the feature table was normalized with the “qiime picrust2 normalize” command, followed by the “qiime picrust2 predict-metagenomes” to predict the metagenome using the software’s built-in reference database. The prediction results were then categorized into Kyoto Encyclopedia of Genes and Genomes (KEGG) Class 2 and Class 3 pathways with “qiime picrust2 categorize-by-function” (51, 52). Each germination stage included 10 biological replicates, and after applying the robust regression and outlier removal (ROUT) method, at least eight data points from each stage should be retained. A Kruskal-Wallis test with Dunn’s test was used to assess the significance of functional differences between stages. Figures were generated using Prism (GraphPad 10.0.0.3).

### Isolation of endophytic *Pseudomonas* bacteria strain

*Pseudomonas* strains were isolated from protocorms (Stages 4 and 5) using a modified *Pseudomonas* cephaloridine fucidin cetrimide (CFC) selective agar, according to Zhang et al. (53). The protocorms were surface sterilized as previously described and then ground into a homogenate by a sterile mortar and mixed thoroughly with 2 mL of sterile water. Next, 200 µL of the homogenate was added to 1 L sterile water, vigorously mixed, and subjected to sequential dilutions using a gradient method (three to five dilutions). Each dilution was plated on 20–50 plates with 50 µL absorbed on each plate. The plates were incubated in darkness at 28°C for 2–3 days until bacterial colonies appeared. Monoclonal colonies were selected and transferred onto new CFC selective plates for streak plate purification. Purification was repeated five to seven times until pure cultures were obtained. Monoclonal colonies were transferred to 450 µL of lysogeny broth (LB) medium. The 16S rRNA gene of each bacterial strain was amplified using 27F (5′-TACGGYTACCTTGTTACGACTT-3′) and 1492R (5′-AGAGTTTGATCMTGGCTCAG-3′) primers, purified and sequenced. The sequences were compared against the NCBI Nucleotide collection online database using BLASTN, and relevant reference sequences, including other *Pseudomonas* strains, were retrieved. The ASV sequences annotated to *Pseudomonas* were aligned with the isolated strains and reference sequences using DNAMAN and MEGA 11 software. A neighbor-joining phylogenetic evolutionary tree was constructed using 10,000 bootstrap replications.

### Effect of *Pseudomonas* species on promoting seed germination and protocorm development

The obtained *Pseudomonas* isolates were incubated for 24 hours at 28°C with shaking at 120 rpm in beef extract peptone medium (BPM), which consisted of 10 g L^−1^ peptone, 3 g L^−1^ beef extract, and 5 g L^−1^ NaCl (pH 7.0). Bacterial cells were obtained by centrifugation and resuspended in a sterile phosphate buffer (10 mM, pH 7.0) to a concentration of about 10^8^ colony-forming units (CFU) mL^−1^. For bacterial inoculation, a 20-µL drop of bacterial suspension was applied to the surface of the oatmeal agar (OMA) medium. The fungus *Ceratobasidium* sp. GS2 was cultivated on PDA media in Petri dishes. The hyphae-containing plugs were excised and transferred to an OMA medium (one plug per dish in the center). Seeds of *G. conopsea* were surface sterilized with 2.5% sodium hypochlorite for 8 minutes, rinsed with sterile water, and then evenly distributed on the surface of the bacteria-inoculated OMA medium.

To investigate the effect of *Pseudomonas* isolates on seed germination, both individually and in the presence of *Ceratobasidium* sp. GS2, we used the following experimental treatments: (i) seeds and *Pseudomonas* isolates (binary coculture); (2) seeds, *Pseudomonas* isolates, and *Ceratobasidium* sp. GS2 (ternary coculture). As control groups, a drop of pure BPM without bacteria was added to the surface of the OMA media containing only seeds or seeds and *Ceratobasidium* sp. GS2. The four unique *Pseudomonas* isolates, labeled P01, P02, P03, and P04, were utilized as bacterial treatment additives. Each Petri dish contained about 200 seeds. Each treatment consisted of six replicates, and the cultures were maintained in darkness at 28°C. The seed germination rate was calculated by dividing the number of germinated seeds by the total number of seeds sown in the Petri dish. The length and width of protocorms were measured using a Zeiss SteREO Discovery stereomicroscope (V12 ProgRes CapturePro v2.8.8 software) after 2 months of inoculation when most protocorms were at Stage 4. We obtained 39, 47, 42, 39, and 42 protocorms, respectively, from the control, P01, P02, P03, and P04 treatment groups. To test the normality of the protocorm sizes within each group, we employed the Shapiro-Wilk test, and the results indicated that the data did not meet the assumption of normality. Consequently, we used non-parametric Kruskal-Wallis tests to evaluate whether germination rates and protocorm sizes significantly differed between treatments. Subsequently, Dunn’s test was performed to assess which of the treatments were statistically significantly different. The results were visualized using Prism (GraphPad 10.0.0.3).

## RESULTS

### Quality metrics for sequencing analysis and assemblies

In total, 331,383 raw circular consensus sequencing (CCS) reads were obtained for bacterial 16S analysis and 3,989,499 raw reads for fungal ITS2 analysis. After trimming and removing chimeric and plant sequences, 324,186 and 3,703,248 high-quality reads were assigned to 1,337 bacterial ASVs and 1,148 fungal ASVs, respectively. The bacterial ASVs were classified into 13 phyla and 192 genera, while fungal ASVs were assigned to 9 phyla and 242 genera (Table S1). Rarefaction analyses demonstrated that the sampling size was sufficient to capture the true diversity of the samples (Fig. S1).

### Alterations in the diversity of endophytic bacteria and fungi throughout protocorm developmental stages

**Fig 2:**
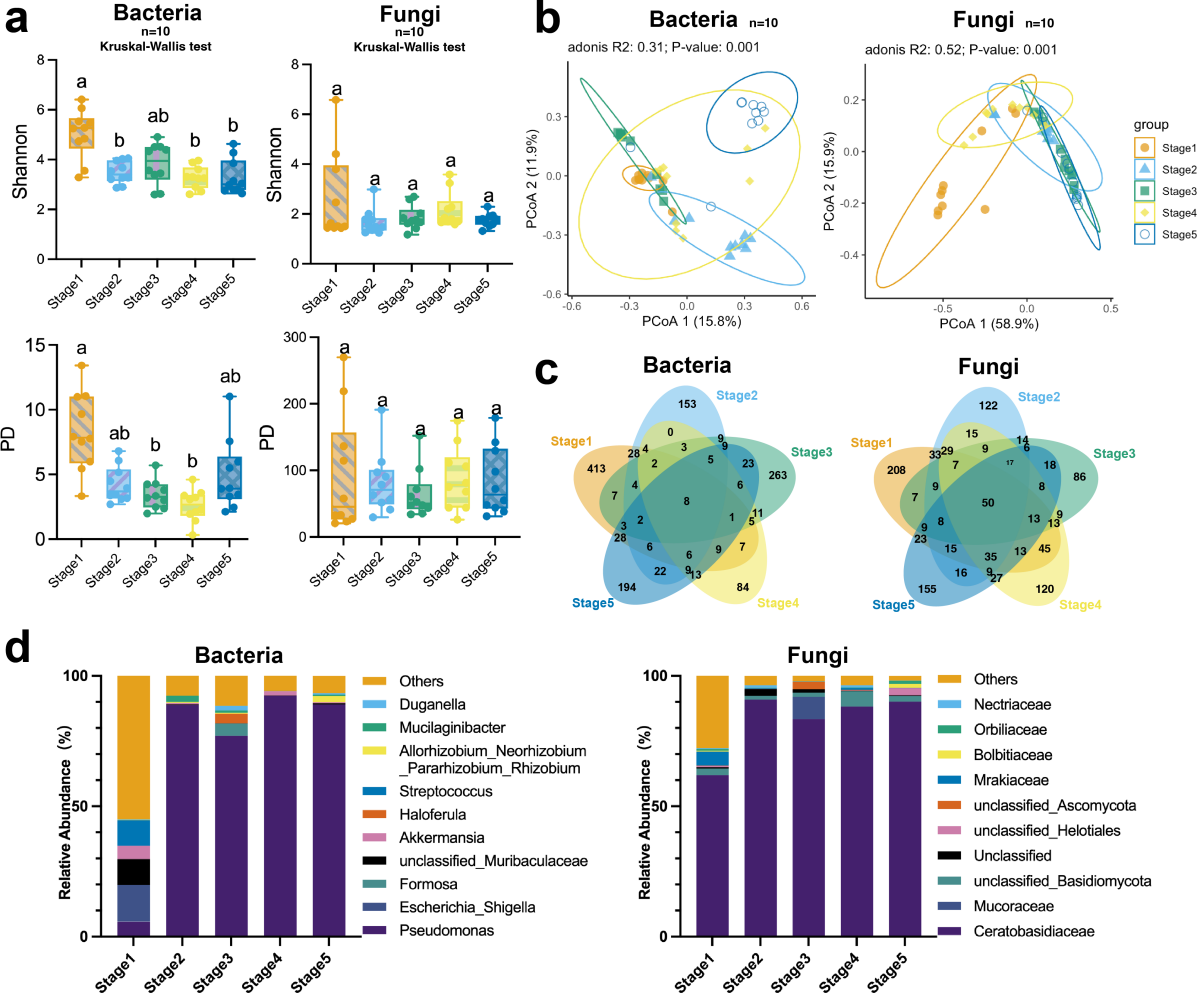
Diversity and community composition of bacterial and fungal communities found in five developmental stages in the germination process of *Gymnadenia conopsea* seeds. (**a**) Assessment of α-diversity (Shannon-Wiener index) and PD of bacterial and fungal communities. (**b**) PCoA analysis shows variation in bacterial and fungal communities between developmental stages. (**c**) Venn diagrams of bacterial and fungal community at ASV level. (**d**) The bar charts illustrate the relative abundance of bacterial and fungal composition between different stages.

For bacterial ASVs, the Shannon index differed significantly among developmental stages, with Stage 1 displaying the highest diversity and Stage 5 exhibiting the lowest (Fig. 2a). Stages 2 (*P* = 0.0365), 4 (*P* = 0.041), and 5 (*P* = 0.017) displayed significantly lower Shannon indices than Stage 1. The PD declined with development stage up to Stage 4, but then recovered in Stage 5. Stages 3 (*P* = 0.0066) and 4 (*P* < 0.0001) showed significantly lower PD values than Stage 1. For fungal ASVs, no significant differences in α-diversity were observed among developmental stages. However, some samples in Stage 1 exhibited higher diversity indexes.

Both bacteria (*R*^2^ = 0.309, *F* = 5.036, *P* = 0.001) and fungi (*R*^2^ = 0.518, *F* = 12.085, *P* = 0.001) showed significant differences in community composition among the five stages (Fig. 2b; Table S2). Pairwise-adonis analyses showed that bacterial communities differed significantly between all compared stages. Except for Stages 2 and 3 (*P* adjusted = 0.109), Stages 2 and 5 (*P* adjusted = 0.069), and Stages 3 and 5 (*P* adjusted = 0.109), fungal communities differed significantly between all other stages.

For bacteria, the number of ASVs found in Stage 1 to Stage 5 individuals was 533, 270, 361, 173, and 344, respectively (Fig. 2c). All five stages shared a core of eight ASVs (Table S3). The number of fungal ASVs detected in Stage 1 to Stage 5 individuals was 517, 394, 283, 419, and 422, respectively. Fifty ASVs were shared in five stages (Table S3). In all stages except Stage 1, *Pseudomonas* was the most abundant bacterial ASV (>75%) (Fig. 2d). Members of Ceratobasidiaceae were identified as the most abundant fungal family across all stages (>60%). ASVs annotated as *Ceratobasidium* sp. GS2 accounted for 83.99% of all the fungal reads.

### Temporal dynamics of endophytic microbiome taxa in protocorms

**Fig 3:**
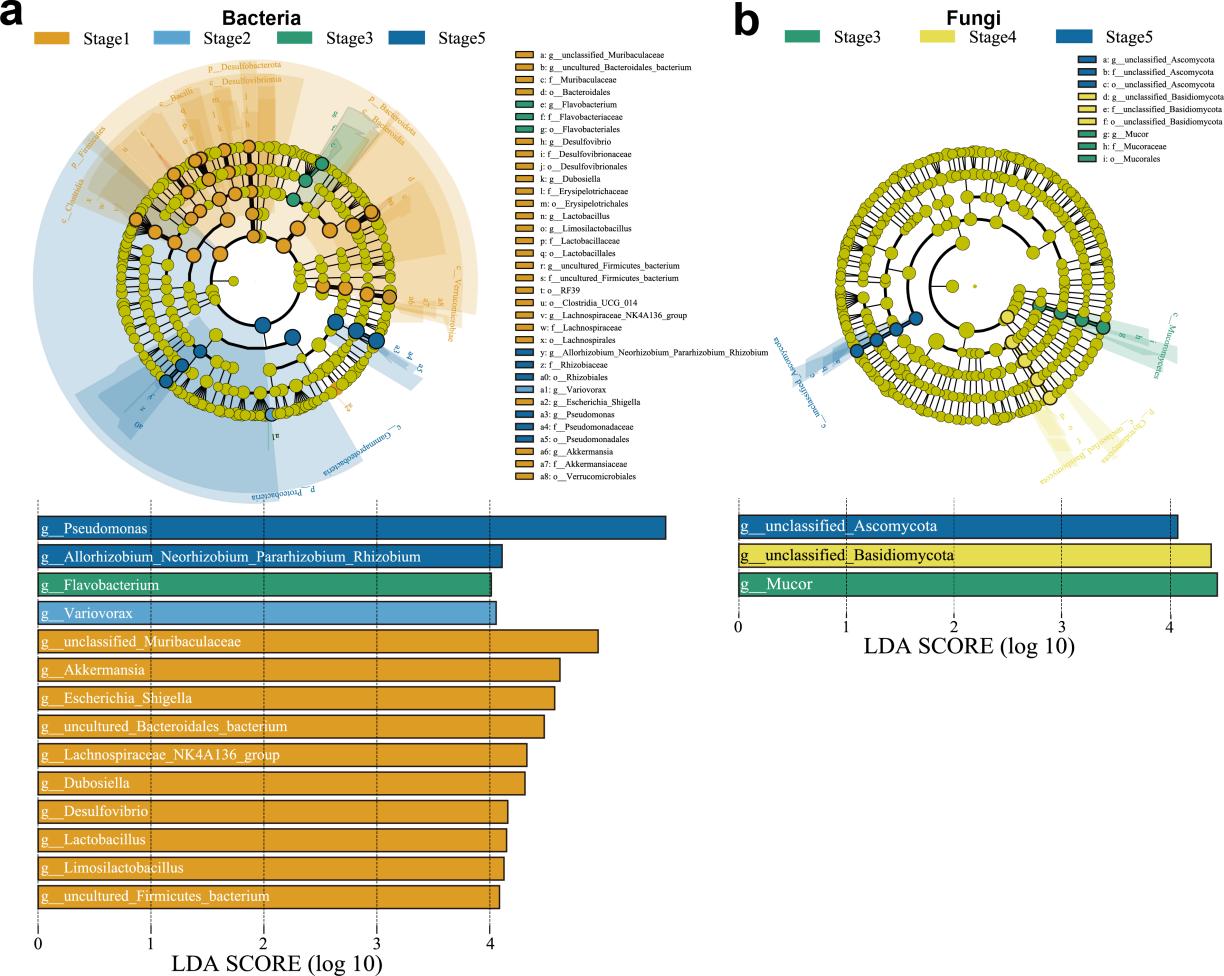
LEfSe analysis of bacterial and fungal taxa. (**a**) Cladograms of bacterial taxa from phyla level to genus level and LDA score histogram (log_10_ > 4.0). (**b**) Cladograms of fungal taxa from phyla level to genus level and LDA score histogram (log_10_ > 4.0).

Fourteen bacterial and three fungal genera were identified as significant contributors to the variations observed among the five developmental stages (Fig. 3a and b). In terms of bacterial biomarkers, Stage 1 exhibited the most biomarkers. Additionally, Stage 5 displayed two biomarkers (*Allorhizobium-Neorhizobium-Pararhizobium-Rhizobium* and *Pseudomonas*). Regarding fungal biomarkers, Stage 3, Stage 4, and Stage 5 each exhibited a distinct biomarker. The absence of particular stages in the figure indicates that no biomarkers passed the screening threshold. The abundance of bacterial ASVs identified as “*Pseudomonas*” showed an increase across Stages 1–5, as indicated by the indicator species analysis (Table S4).

### Co-occurrence networks of bacteria and fungi and identification of keystone taxa

**Fig 4:**
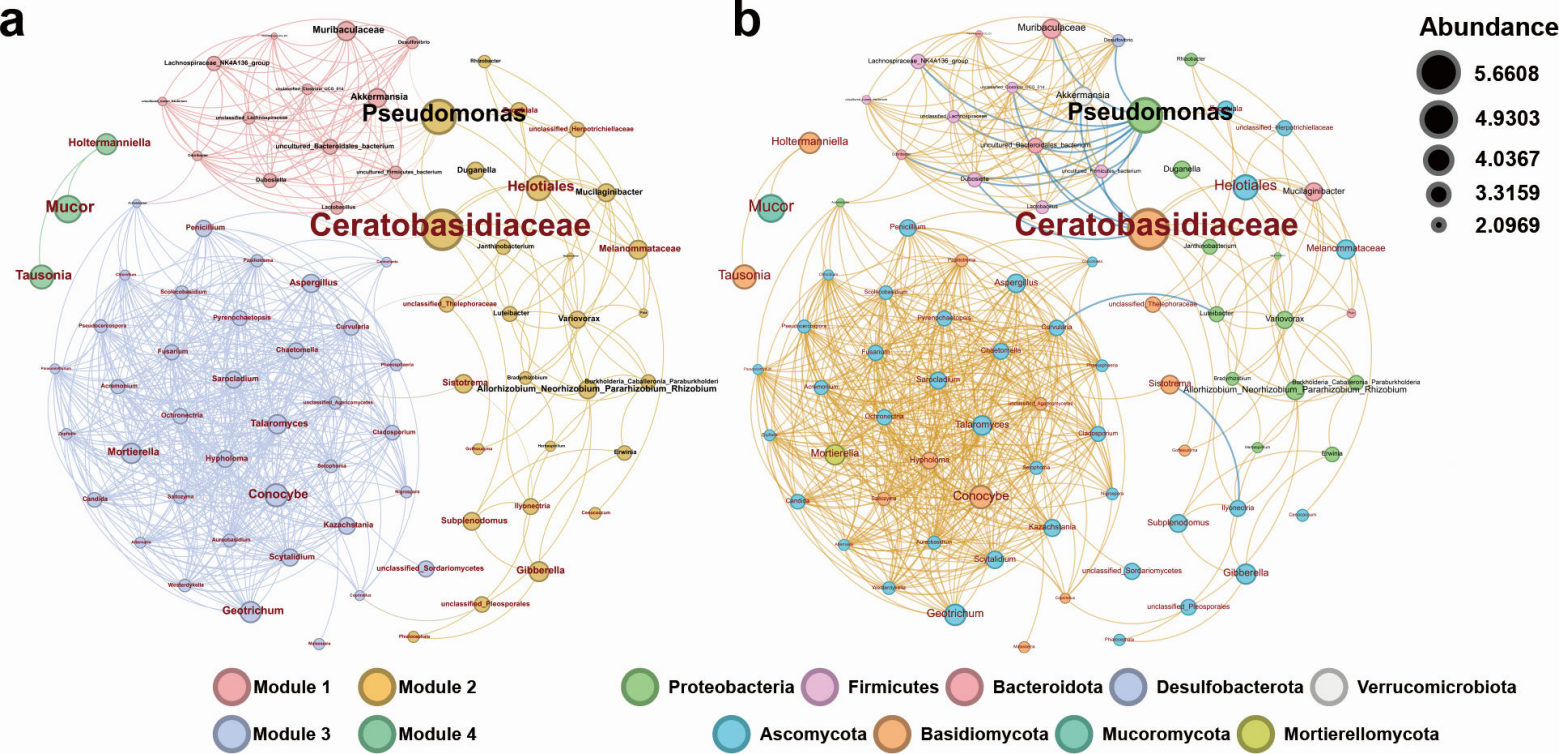
Co-occurrence network analysis at the genus level for both fungi and bacteria. (**a**) The network illustrates the co-occurrence patterns of bacteria and fungi across all developmental stages, with node sizes representing abundance levels. Different colors represent modules. Bacterial communities are labeled in black, while fungal communities are represented in red. (**b**) Co-occurrence network of bacteria and fungi at the phylum level across all developmental stages, with yellow lines denoting positive correlations between nodes, and blue lines representing negative correlations. Node size and font color convey the same information described in (**a**).

The co-occurrence network analysis based on all five stages showed that the bacterial and fungal communities are divided into four modules (Fig. 4a). Module 1 consisted exclusively of bacterial genera, including members of Firmicutes (7), Bacteroidota (4), Desulfobacterota (1), and Verrucomicrobiota (1) (Fig. 4b). Module 2 consisted of a mixture of fungal and bacterial genera, comprising 14 bacterial (12 Proteobacteria and 2 Bacteroidota) and 14 fungal genera (10 Ascomycota and 4 Basidiomycota). Module 3 consisted predominantly of fungi, with only one bacterial genus related to Proteobacteria. It encompassed a total of 37 fungal genera, which were assigned to Ascomycota (29), Basidiomycota (7), and Mortierellomycota (1). The fourth module contained three fungal genera, two belonging to Basidiomycota and one to Mucoromycota. The networks of different stages revealed a gradual decrease in complexity and node correlation and an increase in modularity from Stage 1 to Stage 3 (Fig. S2). From Stage 3 to Stage 4, there was an increase in network complexity but a decrease in modularity, indicating an increasing network chaos (Table S5). Finally, at Stage 5, the modularity index reached its highest value, indicating a highly structured network.

Notably, fungi of the Ceratobasidiceae showed positive correlations with eight nodes belonging to Module 2 (including *Pseudomonas*, *Mucilaginibacter*, *Variovorax*), and negative correlations with five nodes belonging to Module 1 (Fig. 4b). Meanwhile, *Pseudomonas* showed positive correlations with three Module 2-associated genera (including a genus in Ceratobasidiceae, *Variovorax*, and *Mucilaginibacter*), and negative correlations with 11 genera from Module 1.

### Differences in the functional profile of bacterial nutrient metabolism between different stages of protocorm development

**Fig 5:**
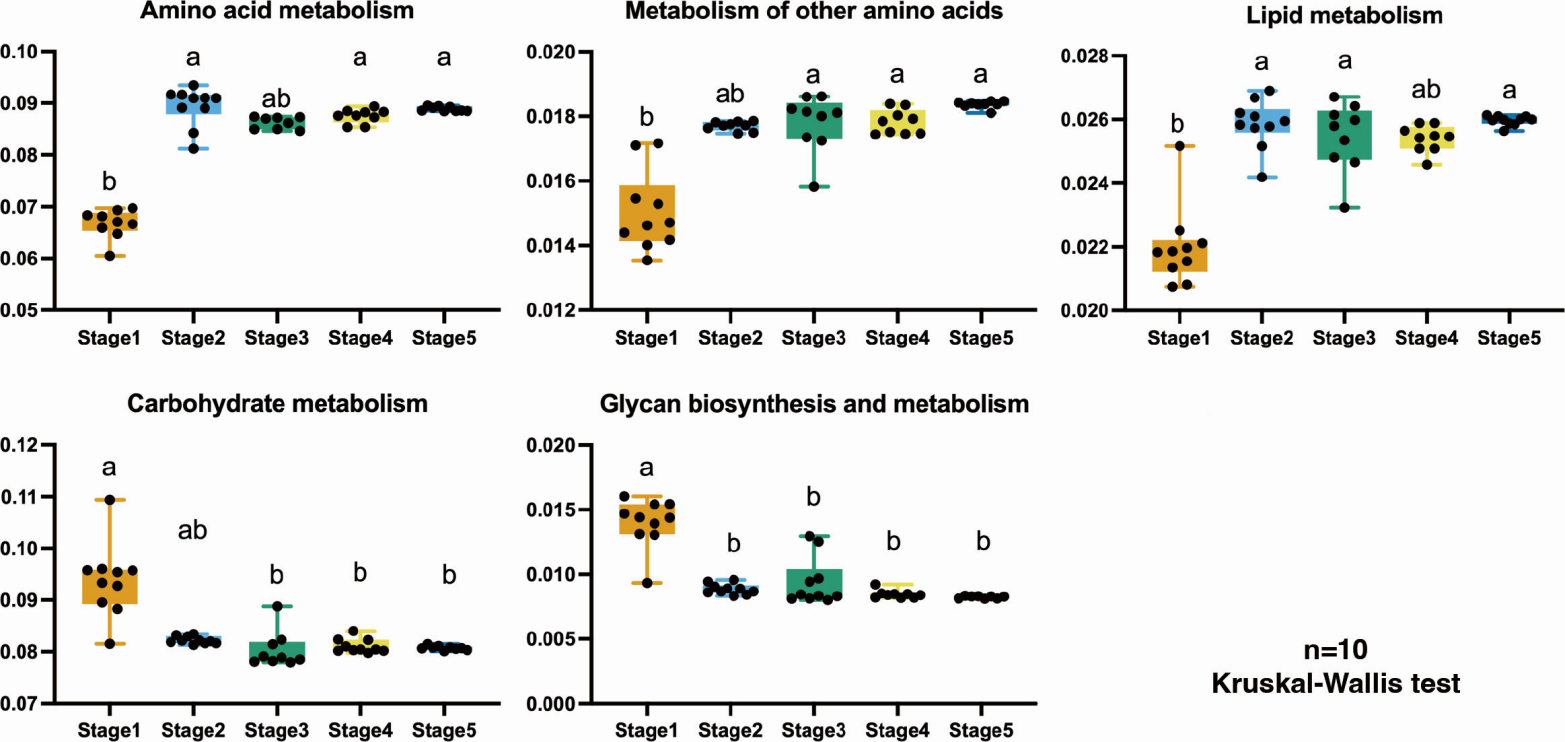
Functional annotation of bacterial groups observed in five stages of protocorm development of the terrestrial orchid *Gymnadenia conopsea* analyzed with PICRUSt2 compared to Class 2 metabolic pathways in the KEGG database. Distinct letters indicate statistically significant differences between developmental stages (Dunn’s test, *P* < 0.05).

PICRUSt2 revealed functional changes related to bacterial nutrient metabolism. At the Class 2 level, germinating seeds (Stage 1) were more distinct from the other four stages (Fig. 5). The distinct functional profile of Stage 1 seeds was consistently observed across the majority of Class 3 level, further confirming their unique functional profile compared to the other stages (Table S6).

Regarding Class 2 pathways “Amino acid metabolism” and “Metabolism of other amino acids,” germinating seeds (Stage 1) showed lower levels than the other stages in general. In contrast, Class 2 pathways associated with “Carbohydrate metabolism” and “Glycan biosynthesis and metabolism” demonstrated higher levels in Stage 1. These include critical metabolic processes such as “Amino sugar and nucleotide sugar metabolism,” “Fructose and mannose metabolism,” “Galactose metabolism,” among others (Table S6). In terms of “lipid metabolism,” we observed a higher activity in pathways related to “Biosynthesis of unsaturated fatty acids” and “Fatty acid degradation” in the last four stages.

### *Pseudomonas* bacteria combined with *Ceratobasidum* fungi promote protocorm growth

**Fig 6:**
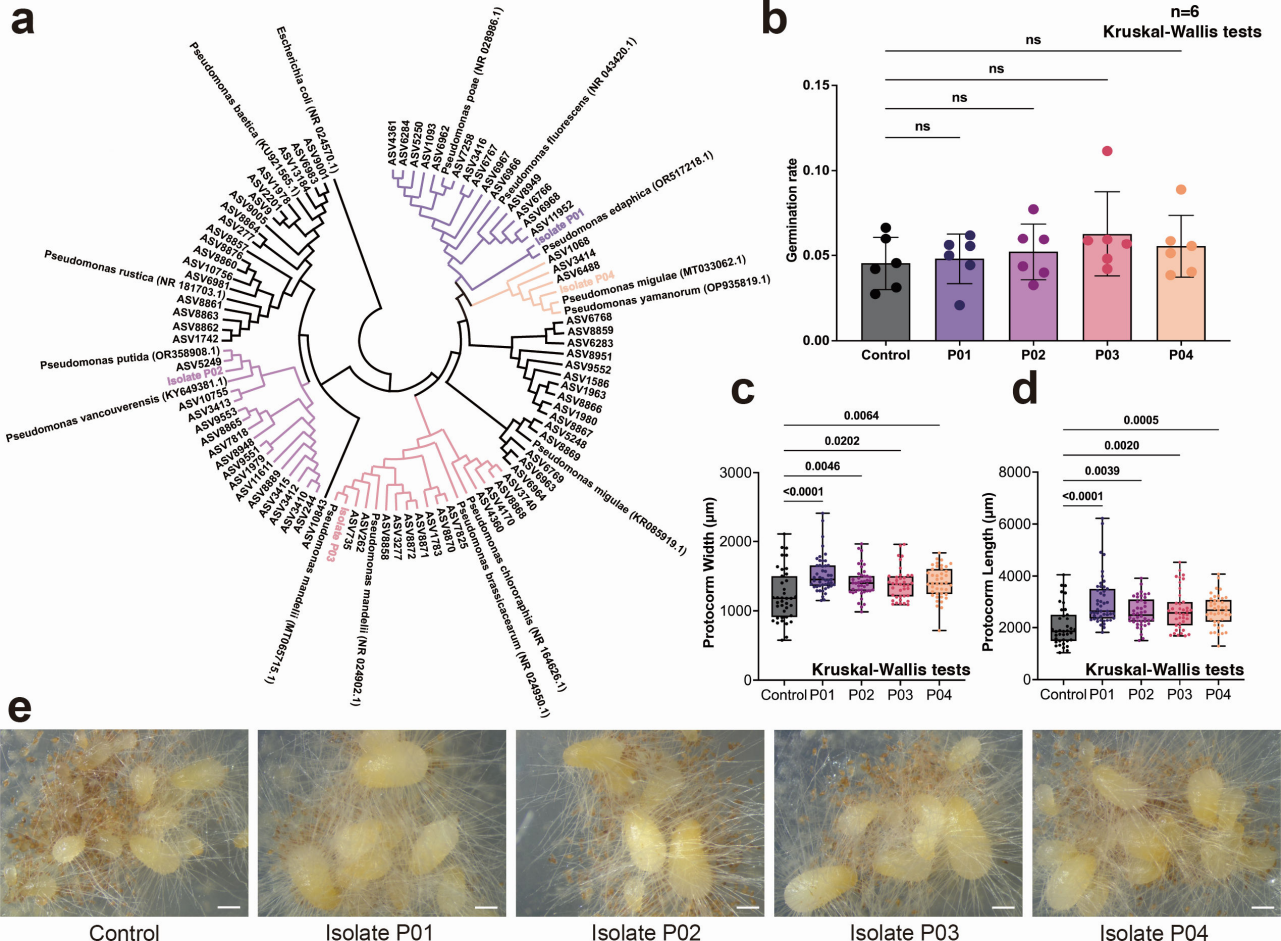
The synergistic effects of four different *Pseudomonas* isolates on seed germination and development in the terrestrial orchid *Gymnadenia conopsea* combined with the fungus *Ceratobasidium* sp. GS2. (**a**) Phylogenetic relationships of the studied bacterial isolates. (**b**) Seed germination rates under coculture conditions involving both bacteria and fungi. ns, not significant. (**c**) Box plots showing differences in protocorm length. Dunn’s test was utilized to evaluate the statistical significance of the disparity between the treated and control groups, with the corresponding *P* values presented. (**d**) Box plots showing differences in protocorm width. (**e**) Micrographs of cocultured protocorms treated with various *Pseudomonas* isolates (scale bar = 1,000 µm).

Altogether, 44 *Pseudomonas* bacterial isolates belonging to four strains were obtained and grouped into distinct clades (Fig. 6a). However, none of them was able to induce seed germination without the fungus *Ceratobasidium* sp. GS2. Consequently, all results presented below are from the ternary coculture experiments containing seeds, fungi, and the bacteria. After 2 months of culturing, we obtained 39, 47, 42, 39, and 42 protocorms in the control, P01, P02, P03, and P04 treatment groups. The four strains did not significantly enhance germination rates compared to the control group (Kruskal-Wallis test: *P* = 0.8017) (Fig. 6b). However, the size of protocorms was significantly larger in the *Pseudomonas* treatments than in the control treatment (Dunn’s test: *P* values are shown in the figure, Fig. 6c and d). Visual inspection reveals that the protocorms show significantly larger volumes when bacteria are added to the media, than those in the control group (Fig. 6e).

## DISCUSSION

Bacteria and fungi commonly co-occur in the soil, and their interactions play an essential role in various ecosystem functions, including biogeochemical cycling, plant production, and mitigation of potential pathogenic risks (11). In plants associated with mycorrhizal fungi, bacteria have been shown to stimulate the growth and spore development of arbuscular mycorrhizal fungi and further enhance plant growth and development (12, 13, 54, 55). However, the role of bacteria in orchid mycorrhizal symbiosis, especially in orchid seed symbiotic germination, remains largely unknown.

In this study, we used *in situ* germination experiments to obtain protocorms of the terrestrial orchid *G. conopsea* at various developmental stages (from germinating seeds to fully developed protocorms with the appearance of the first leaf) and to evaluate the bacterial and fungal communities associating with these different developmental stages. A diverse bacterial community was detected across the different germination stages, with members of Proteobacteria being the most abundant phylum overall. *Pseudomonas* showed the highest relative abundance at the genus level, except in germinating seeds (Stage 1). Compared to the other four stages, the bacterial community in germinating seeds was significantly different. The relative abundances of dominant bacteria decrease significantly in the following stages, indicating those bacteria are more opportunistic or pathogenic microorganisms that do not play an essential role in germination. In the comprehensive co-occurrence network covering all five developmental stages, Ceratobasidiceae fungi and *Pseudomonas* bacteria negatively correlated with bacterial groups in germinating seeds, suggesting their potential resistance to pathogenic bacteria. Similar enhanced tolerance to abiotic stress and antagonistic activity against pathogens have been observed in endophytic bacteria and fungi (56–59).

Interactions within root-associated microbiomes can enhance host nutrient acquisition and promote plant fitness (60, 61). This research identified a potential synergistic relationship between a dominant member of the fungal family Ceratobasidiaceae and several *Pseudomonas* bacteria during protocorm development. We observed a positive correlation between these strains in the co-occurrence network and a decrease in network complexity and stability when seeds developed from Stage 1 to Stage 5. Previous research has shown that highly connected networks often form in response to environmental stresses such as nutrient scarcity and pathogen invasion (62, 63). The observed decline in network complexity and stability during protocorm development suggests a reduced capacity to mitigate nutrient stress through intensive metabolic interactions.

Furthermore, functional annotation revealed significant differences in functional profiles between bacterial groups in Stage 1 and later developmental stages (Stages 2–5). Specifically, there was a gradual increase in lipid and amino acid metabolism, while carbohydrate metabolism showed a significant decrease. This observation aligns with the fact that orchid seeds possess substantial reserves of fatty acids and proteins, necessitating the utilization of carbohydrates as carbon sources for germination (64–67).

Among these beneficial microbes, *Pseudomonas* spp. are known as plant growth-promoting bacteria (PGPB) that interact beneficially with plants by colonizing the rhizosphere and various compartments of plants. *Pseudomonas* spp. are phylogenetically diverse and exhibit a wide range of metabolic and ecological functions, such as stimulating plant growth by facilitating nutrient uptake, regulating phytohormone levels, and offering direct and indirect biocontrol of phytopathogens through induced systemic resistance (68). Therefore, *Pseudomonas* spp. have been widely used as biological bacterial fertilizers (69–71). Our results showed a significant increase in the abundance of *Pseudomonas*, ultimately becoming the dominant bacterial taxa in the later stages of protocorm development. This suggests that *Pseudomonas* bacteria contribute to symbiotic germination in orchids and that the plants or fungi actively recruited them. In line with this hypothesis, our results showed that *Pseudomonas* emerged as a distinctive biomarker for fully developed protocorms (Stage 5), which may be indicative of their involvement in facilitating the germination process, particularly growth to seedlings (31, 72, 73). Therefore, isolating bacterial strains from developing protocorms and applying them in cultivation or restoration programs could be beneficial for enhancing protocorm development.

Interestingly, *Pseudomonas* isolates significantly enhanced protocorm growth only in the presence of the fungus *Ceratobasidium* GS2. It remains unclear whether protocorms directly profited from the *Pseudomonas* bacteria or through interactions between *Pseudomonas* and *Ceratobasidium* GS2. Recent research has shown that bacteria can exert direct plant growth-promoting effects or function indirectly as a mycorrhizal helper bacteria (MHB) (12). MHB enhance the growth of mycorrhizal fungi through the secretion of branching factors that regulate actin cytoskeleton dynamics within fungal hyphae, thereby promoting hyphal extension (13, 74). They can also provide nutrients such as synthetic vitamins or compounds to support the growth of mycorrhizal fungi (54, 75). On the other hand, recent genome analyses of many plant-associated *Pseudomonas* strains have shown they possess growth-stimulating traits (76). Hence, the exact mechanisms leading to increased protocorm growth still need to be clarified in future studies.

Overall, we conclude that co-inoculation of bacteria and fungi had a positive impact on the growth of mycorrhizal-dependent heterotrophic protocorms of *G. conopsea*. These findings indicate that bacteria exert non-negligible effects on seed germination of orchids and, therefore, offer valuable perspectives for future strategies for conservation and cultivating orchid species. Further research is needed to better understand the mechanisms underlying the interactions between bacteria, orchids, and fungi. Additionally, exploring in more detail the synergistic relationships between orchid root-associated bacteria and mycorrhizal fungi holds great potential for enhancing orchid cultivation and the design of more effective management and conservation practices in natural habitats.

## Data Availability

PacBio sequencing data have been deposited with BioProject accession number PRJNA1098998 in the NCBI database.

## SUPPLEMENTAL MATERIAL

Supplemental material spectrum.02185-24-s0001.docx Fig. S1 and S2.

Table S1 spectrum.02185-24-s0002.xlsx ASV counts and taxonomy were generated using DADA2 for bacterial 16S rRNA sequencing.

Table S2 spectrum.02185-24-s0003.xlsx PERMANOVA and pairwise-Adonis results of bacteria.

Table S3 spectrum.02185-24-s0004.xlsx The bacterial core ASVs shared by all five stages.

Table S4 spectrum.02185-24-s0005.xlsx The bacterial and fungal indicator ASVs in five protocorm stages (indicator value > 0.5, *P* < 0.05).

Table S5 spectrum.02185-24-s0006.xlsx Overview of the main topological characteristics of the co-occurrence network at each developmental stage.

Table S6 spectrum.02185-24-s0007.xlsx The PICRUSt2-annotated nutrient metabolism pathways that exhibit significant differences across five stages.

ASM does not own the copyrights to Supplemental Material that may be linked to, or accessed through, an article. The authors have granted ASM a non-exclusive, world-wide license to publish the Supplemental Material files. Please contact the corresponding author directly for reuse.
