# Supplementary material for: Shifts in bacterial community composition during symbiotic seed germination of a terrestrial orchid and effects on protocorm development
Source: Microbiol Spectr. 2024 Nov 14;12(12):e02185-24. doi: 10.1128/spectrum.02185-24 (PMC11619447; doi:10.1128/spectrum.02185-24)
Supplement: Supplemental material — Fig. S1 and S2. [file spectrum.02185-24-s0001.docx]

**Supplemental Information**

**Shifts in bacterial community composition during symbiotic seed germination of a terrestrial orchid and effects on protocorm development**

Zeyu Zhao^1#^, Luna Yang^1#^, Yaoyao Wang^1^, Xin Qian^1^, Gang Ding^1^, Hans Jacquemyn^2^, Xiaoke Xing^1^*

**Author affiliations**

^1^State Key Laboratory for Quality Ensurance and Sustainable Use of Dao-di Herbs, Institute of Medicinal Plant Development, Chinese Academy of Medical Sciences and Peking Union Medical College, Beijing, China

^2^Department of Biology, Plant Conservation and Population Biology, Katholieke Universiteit Leuven, Leuven, Belgium

# These authors share the first authorship.

*Correspondence

Xiaoke Xing, Email: xkxing2009@hotmail.com

Email address for each author

Zeyu Zhao [zyzhao2017@163.com](mailto:zyzhao2017@163.com)

Luna Yang [1010337215@qq.com](mailto:1010337215@qq.com)

Yaoyao Wang 18810235913@163.com

Xin Qian qx731592766@163.com

Gang Ding gding@implad.ac.cn

Hans Jacquemyn hans.jacquemyn@kuleuven.be


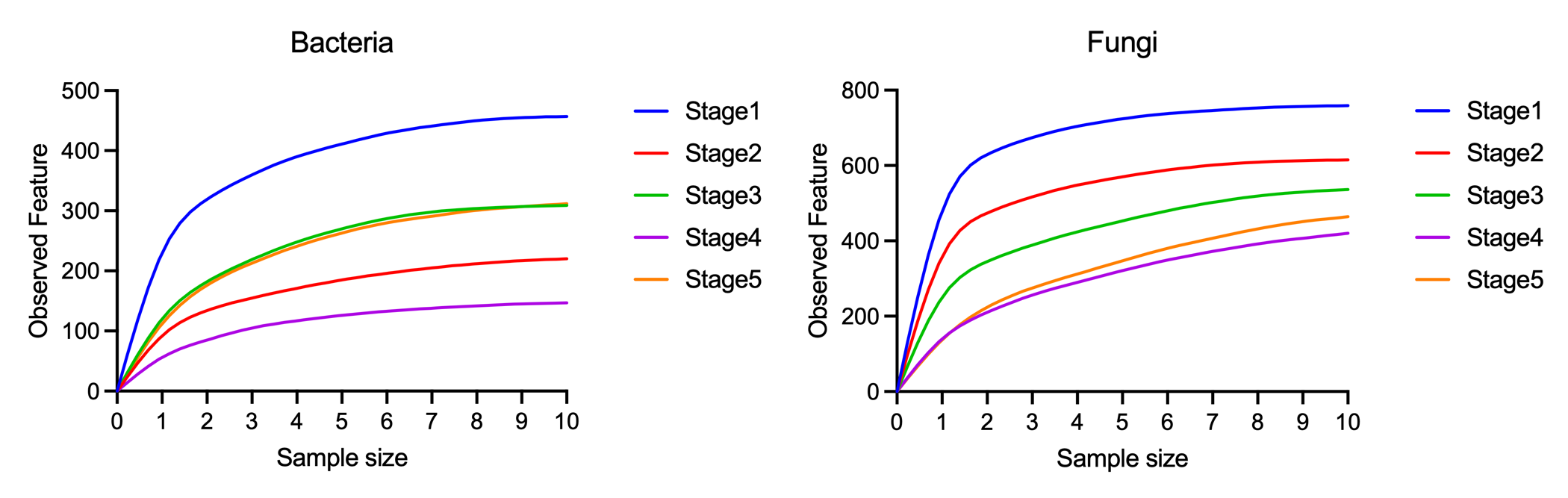


**Figure S1** The sample-based rarefaction curves for bacterial and fungal communities.

**
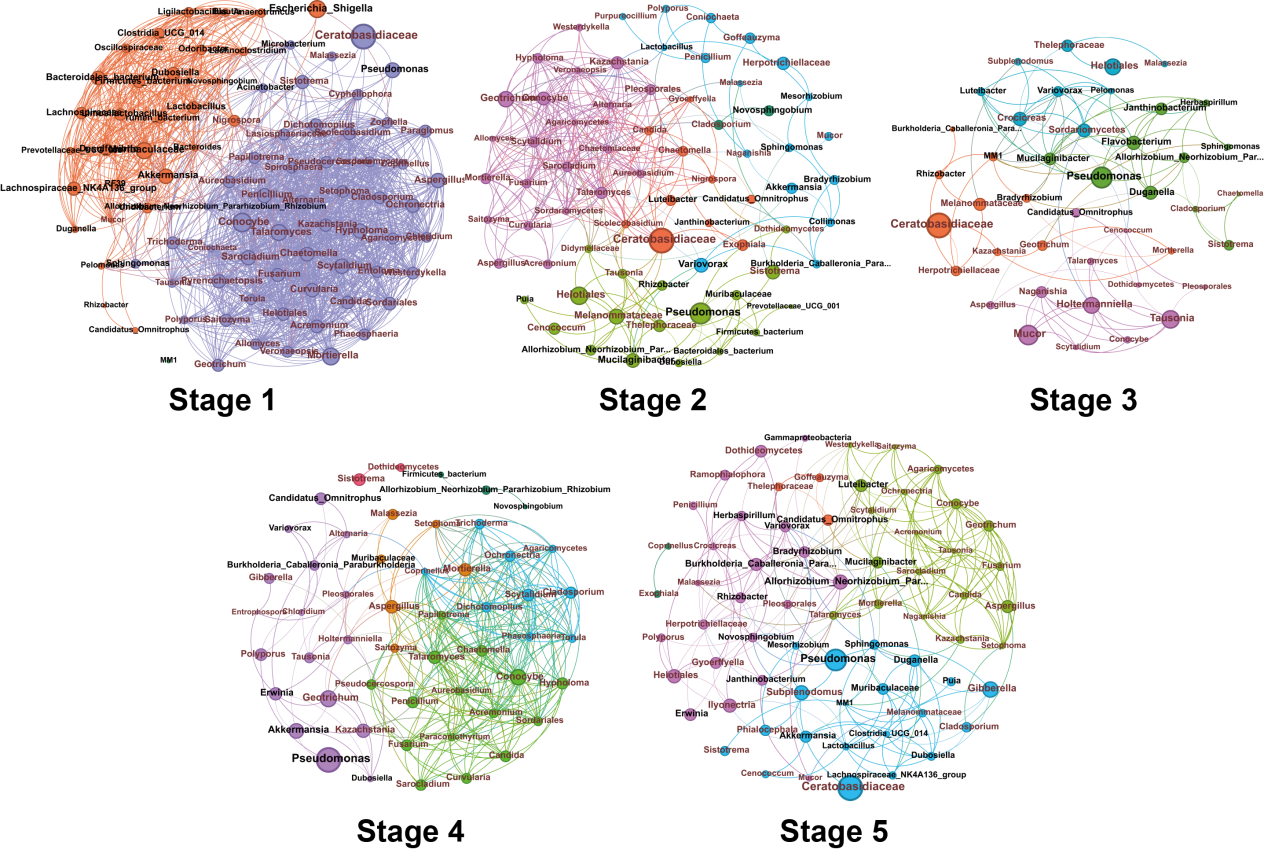
**

**Figure S2.** The co-occurrence network analysis of five protocorms development stages. Bacterial communities were labeled in black, while fungal communities were represented in red.
